# *Achromobacter xylosoxidans* and *Stenotrophomonas maltophilia*: Emerging Pathogens Well-Armed for Life in the Cystic Fibrosis Patients’ Lung

**DOI:** 10.3390/genes12050610

**Published:** 2021-04-21

**Authors:** Quentin Menetrey, Pauline Sorlin, Estelle Jumas-Bilak, Raphaël Chiron, Chloé Dupont, Hélène Marchandin

**Affiliations:** 1 HydroSciences Montpellier, CNRS, IRD, Univ Montpellier, 34093 Montpellier, France; quentin.menetrey@umontpellier.fr (Q.M.); pauline.sorlin@umontpellier.fr (P.S.); 2 HydroSciences Montpellier, CNRS, IRD, Univ Montpellier, Department d’Hygiène Hospitalière, CHU Montpellier, 34093 Montpellier, France; estelle.bilak@umontpellier.fr (E.J.-B.); chloe.dupont@umontpellier.fr (C.D.); 3 HydroSciences Montpellier, Université de Montpellier, CNRS, IRD, Centre de Ressources et de Compétences de la Mucoviscidose, CHU de Montpellier, 34093 Montpellier, France; r-chiron@chu-montpellier.fr; 4 HydroSciences Montpellier, CNRS, IRD, Univ Montpellier, Service de Microbiologie et Hygiène Hospitalière, CHU Nîmes, 34093 Nîmes, France; 5 UMR 5151 HydroSciences Montpellier, Equipe Pathogènes Hydriques Santé Environnements, U.F.R. des Sciences Pharmaceutiques et Biologiques, Université de Montpellier, 15, Avenue Charles Flahault, BP 14491, CEDEX 5, 34093 Montpellier, France

**Keywords:** emerging pathogens, cystic fibrosis, *Stenotrophomonas*, *Achromobacter*, diversity, pathoadaptation, virulence, resistance, competition, persistence

## Abstract

In patients with cystic fibrosis (CF), the lung is a remarkable ecological niche in which the microbiome is subjected to important selective pressures. An inexorable colonization by bacteria of both endogenous and environmental origin is observed in most patients, leading to a vicious cycle of infection–inflammation. In this context, long-term colonization together with competitive interactions among bacteria can lead to over-inflammation. While *Pseudomonas aeruginosa* and *Staphylococcus aureus*, the two pathogens most frequently identified in CF, have been largely studied for adaptation to the CF lung, in the last few years, there has been a growing interest in emerging pathogens of environmental origin, namely *Achromobacter xylosoxidans* and *Stenotrophomonas maltophilia*. The aim of this review is to gather all the current knowledge on the major pathophysiological traits, their supporting mechanisms, regulation and evolutionary modifications involved in colonization, virulence, and competitive interactions with other members of the lung microbiota for these emerging pathogens, with all these mechanisms being major drivers of persistence in the CF lung. Currently available research on *A. xylosoxidans* complex and *S. maltophilia* shows that these emerging pathogens share important pathophysiological features with well-known CF pathogens, making them important members of the complex bacterial community living in the CF lung.

## 1. Introduction

Cystic fibrosis (CF) is the most commonly, still fatal, inherited genetic disease in Caucasian populations, caused by a mutation in the cystic fibrosis transmembrane conductance regulator (CFTR) gene coding for a transmembrane channel allowing the transport of chloride ions. Different organs are affected, including the respiratory tract, where thick mucus, mucociliary clearance defects, and a decrease in anti-microbial defenses favoring bacterial colonization are observed [1]. The main causes of morbidity and mortality in CF patients are recurrent respiratory infections. From early childhood, colonization is often initiated by *Staphylococcus aureus* or *Haemophilus influenzae*. Later, the microbiology of the lung becomes more complex, as *Pseudomonas aeruginosa* becomes dominant, and the lung may also be colonized by other microorganisms such as nontuberculous mycobacteria (NTM), *Burkholderia cepacia* complex, *Achromobacter xylosoxidans,* and *Stenotrophomonas maltophilia* [2,3].

## 2. Emerging Pathogens and Colonization of the Lungs of Cystic Fibrosis Patients

*Sm*, *Axc,* and NTM are considered as emergent in CF [2] based on the increase in patients colonized over 20 years reported in the French CF registry (10.3% of the patients in 2018 versus 4.7% in 1999 for *Sm*, 6.7% versus 3.1% for *Axc,* and 3.1% versus 0.5% for NTM) [4]. Similar trends were reported in the American CF registry [5].

Hereafter, the review has been limited to the aerobic, non-fermentative, Gram-negative rods, ubiquitously distributed in moist environments and soil, *Achromobacter xylosoxidans* and *Stenotrophomonas maltophilia*. We will refer to *Achromobacter xylosoxidans* complex (*Axc*) to consider *Achromobacter xylosoxidans* sensu stricto and related species identified in CF whose distinction requires specific molecular-based methods. However, although *Stenotrophomonas maltophilia* (*Sm*) was also considered as a complex due to its large genetic diversity and its genetic organization in genogroups, we will refer to *S. maltophilia* as the speciation status of the sub-lineages within *S. maltophilia* has still to be clarified [6].

The determinants for emergence are many and varied, related to patient management and the intrinsic characteristics of bacteria [7]. First, emerging pathogens have been better recognized over time, with better patient follow-up, greater life expectancy, an increased use of aggressive eradication therapy for initial infection with “typical” CF pathogens like *P. aeruginosa,* and advances in bacterial identification methods in microbiology laboratories; second, the bacterial characteristics, including significant innate and acquired antimicrobial resistance, as well as their ability to implement various adaptive strategies, contribute to their persistence in the patients’ respiratory tract.

While the clinical impact of *P. aeruginosa* and *S. aureus*, particularly methicillin-resistant *S. aureus*, is proven in the colonization and infection of CF patients, the clinical significance of *Axc* and *Sm* in CF has been a matter of debate, despite the fact that both of them are well-known opportunistic pathogens causing a variety of severe infections (bacteremia, pneumonia, endocarditis, peritonitis, etc.), especially in immunocompromised hosts and patients with underlying diseases [8,9,10,11,12,13]. The implication of *Axc* in the decline of CF lung function is still controversial, although *A. xylosoxidans* has been shown to induce a level of inflammation similar to that caused by *P. aeruginosa* in chronically infected patients [14]. So far, large multicenter studies have yet to decipher the clinical burden of *Axc* colonization, taking into consideration variability factors of previous small cohort studies, i.e., *Axc* species involved, age at first colonization, type of colonization, definition of chronic colonization, and co-colonizer microorganisms [14,15,16,17]. However, one retrospective case-control study on 36 adult patients in France has demonstrated that *Axc* isolation (at least one sputum culture positive for *Axc*) is associated with a greater decline in respiratory function and a higher number of exacerbations compared with control CF patients uninfected by *Axc* [17]. In a study on 34 Canadian adult patients, Edwards et al. also found that patients were more likely to experience pulmonary exacerbation at the time of *Axc* isolation [16]. A focus on patients with chronic colonization showed that the 23 French patients included in the study by Tetard et al. had more exacerbations than intermittently colonized patients in the 3 years after *Axc* colonization [17], a finding not observed in other studies including 15 and 10 chronically colonized patients showing no long-term significant difference in lung function [14,16], except for one subgroup of patients with a rapid increase in *Axc*-specific antibodies [14]. For *Sm*, an increased rate of pulmonary exacerbations and hospitalizations has been demonstrated in colonized CF patients [18,19,20] but the decline in lung function has been differentially observed according to the studies [18,19,21]. The species also elicited significant in vitro (airway epithelial and macrophage cells) and in vivo (BALB/cJ mice) inflammatory response, suggestive of its contribution to patient airway inflammation [22,23]. Nevertheless, *Axc* and *Sm* are carefully considered and treated accordingly by CF clinicians, with both pathogens being recognized as markers of more severe lung disease in affected patients [17,24] and sharing important pathophysiological features with the emblematic CF pathogen, *P. aeruginosa*.

After initial colonization, the success of a strain’s persistence can vary depending on whether its colonization is sporadic, intermittent, or chronic. Chronic colonization is usually defined as the isolation of the bacterial species in more than 50% of sputum samples collected during the last 12 months, with a minimum of four sputum samples analyzed during that period [25]. Persistence of opportunistic environmental bacteria is a frequent observation in the airways of CF patients. In 2018, the French CF registry reported 21% of patients with chronic *P. aeruginosa* colonization [26]. Various other Gram-negative bacilli of environmental origin have also been isolated in CF patients over long periods of time, up to the death of the patient, e.g., *Burkholderia cepacia* complex, *Pandoraea* sp., *Inquilinus* sp., etc. [27,28,29]. For *Axc*, chronic colonization was reported in 11% to 30% of CF patients after the initial colonization [30]. To our knowledge, no similar data are available for *Sm* yet, despite its recognized ability to persist in the CF lung. However, a recently published monocentric study has suggested that intermittent *Sm* colonization is dominant in CF patients, with lower rates of chronically colonized patients (about 3%) observed in this study [31]. During initial colonization, and more markedly during persistence, emerging pathogens are subjected to various significant selective pressures in the lungs of CF patients, just like other members of the CF lung microbiota. CF lung is indeed a remarkable ecological niche in which biotic selective pressures (interactions among microorganisms living within the same ecosystem or with the host immune response) and abiotic selective pressures (non-living factors such as high levels of antibiotic use, modified acidity and salinity of the surrounding environment, oxygen deprivation in mucus, altered antimicrobial peptide production, etc.) continually shape the microbial community.

Based on the recent interest in emerging pathogens in CF and the aforementioned characteristics of *Axc* and *Sm*, the aim of this review is to gather the current knowledge on the major bacterial pathophysiological traits of *Axc* and *Sm* involved in colonization, virulence, and competitive interactions with other members of the lung microbiota, their supporting mechanisms, regulation, and evolutionary modifications during persistence in the airways of CF patients. A focus is made on biofilm biosynthesis, motility, antimicrobial resistance, secretion systems, and diffusible virulence or signal factors, competitive interactions impacting the above parameters, and genotypic adaptive modifications (gene expression, hypermutation, and microevolution). Indeed, all these abilities are considered as major drivers of pathoadaptation and persistence in the CF lung [32]. As largely reported for the “Swiss army knife” *P. aeruginosa* [33,34], this review highlights that the two emerging pathogens under consideration are well-armed for life in the lungs of CF patients, no doubt a key for their success in this setting.

## 3. Hypermutation and Genomic Within-Host Evolution

### 3.1. Hypermutation

Hypermutability is just one of the ways in which bacteria adapt to the diverse surrounding pressures in the CF lung. Due to a deficiency in DNA mismatch repair (nonsynonymous mutations in *mutS*, *mutL*, *mutH*, and/or *uvrD* genes) that provokes an even higher rate of mutation, hypermutation leads to increased bacterial adaptability.

Well-characterized in *P. aeruginosa*, only a few works have investigated hypermutation in *Axc* [35,36,37] and *Sm* [38,39,40,41]. These studies found strains that undergo mutations at a high rate (mutation frequency ≥ 4.0 × 10^−7^) in both *Axc* and *Sm* CF populations and considered hypermutation as a key adaptive pathway of both pathogens.

*Axc* and *Sm* hypermutators have been shown to present distinct alterations of DNA mismatch repair (MMR) with a large deletion in the *mutS* gene demonstrated in two independent studies on *Axc* [36,37] and a H683P amino acid change observed in the *Sm* MutS protein, which impaired its activity [38]. Hypermutation was prevalent among CF clinical isolates of *Axc*, being observed in 44% of the *Axc* isolates (40 of 90 isolates) and 26% of the patients (11 of 42 patients) [36]. The hypermutator phenotype was exclusively associated with chronic infections and over-represented in strains from the Danish epidemic strain of *Achromobacter ruhlandii* [36]. For *Sm*, 31% of CF isolates were hypermutators (28 of 90 isolates) in the studies of Vidigal et al. and Esposito et al., and 16.7% in the study of Turrientes et al. [38,39,40]. However, these percentages must be considered with caution as studies usually included several strains per patient or clonally related strains. One study of 174 *Sm* isolates from clinical (48 strains from 13 CF patients and 66 from 53 non-CF patients) and environmental (60 strains) origins showed that hypermutators were exclusively observed among clinical isolates, mainly originating from CF patients (8 of 48 isolates, 16.7%) compared with non-CF patients (2 of 66 isolates, 3%) [38]. This result indicates that clinical environments might select bacterial populations with high mutation frequencies. Finally, significantly higher mutation frequencies were observed for Sm strains of Sequence Type 184 compared with other genotypes, suggesting that some genetic backgrounds may be more prone to hypermutation [40]. Hypermutation was thus differentially observed among strains and species of *Axc* and *Sm*, but also among clonally related adaptive variants in a patient or in a sample, as isolates presenting different mutation frequencies were shown to coexist within a patient.

Being hypermutable can confer a transient advantage to fluctuating environmental conditions, but mutation status usually evolves over time to a less mutator phenotype due to fitness cost associated with hypermutation [39,41]. However, studies have reported CF patients with successively recovered isolates displaying increased mutation frequencies during persistence [38,40,42], suggesting that the dynamics of hypermutators also depend on specific conditions linked to the host/strain partnership and that the fitness cost of hypermutable Sm strains may not systematically impede long-standing CF lung infection.

### 3.2. Diversified Populations and Within-Host Genome Evolution

The genomes of *Axc* and *Sm* are highly dynamic, and numerous alterations in the genomic content of *Axc* and *Sm*, named microevolution, have been demonstrated during their persistence in the CF lung, as previously studied in *P. aeruginosa* [43,44]. During *Acx* or *Sm* persistence, studies based on pulsed-field gel electrophoresis (PFGE) first led to the recognition of “true” chronic colonization as well as intra-clonal evolution of PFGE profiles over time through genome rearrangements [15,39,40,41,42,45]. As for *P. aeruginosa*, microevolution was related to lung habitat pathoadaptation by the authors. Further investigation of the within-host evolution through the analysis of whole genome sequences of clonal lineages of *Axc* showed that microevolution occurred through small mutations in a high variety of genes, mostly in genes involved in the general metabolism but also in genes involved in virulence, antimicrobial resistance, transcription and translation, cell wall, and stress response [46,47,48]. In *Sm*, genes identified as undergoing adaptive evolution were found to be involved in virulence and resistance to antibiotics and heavy metals [49].

Another level of complexity was brought to light by the discovery of highly complex *Axc* and *Sm* populations composed of a variety of sub-clonal variants, so-called diversified populations, in a patient or sputum sample [40,42,45]. Again, these observations were confirmed by whole genome sequence-based studies showing that a wide majority of mutations were isolate-specific within a clonal lineage in *Axc* [46]. Divergent evolution forming several subpopulations from the original infecting isolate had previously been observed in *P. aeruginosa* and was linked to the heterogeneity of the CF lung, wherein bacteria are submitted to distinct selective pressures leading to distinct evolutionary processes [44]. Here, we would like to elucidate a broad study analyzing 552 *Sm* genomes from 23 sites of the lungs of a CF patient that led to the most complete description of a bacterial population diversified into multiple coexisting lineages for an emerging pathogen in CF [49]. In this study, lineage-separating mutations were found in genes known to be important for virulence. The authors identified mutations arising independently in lineages that colocalize in space, providing evidence for location-specific selection. This also suggested that certain genotypes can locally proliferate because of site-specific selection and that such site-specific selection might contribute to long-term maintenance of diversity in CF chronic infections that could impede therapy [49].

Microevolution thus leads to an important genotypic heterogeneity related to an equally important phenotypic diversity. We previously conducted a study on multiple *Axc* isolates from a sputum sample of several chronically colonized CF patients and observed significant genomic diversity in PFGE associated with a striking diversity in antimicrobial resistance patterns, not predictable from colonial morphology, that may contribute to compromise the antibiotic management of CF airway infections by *Axc* [45]. Similar observations are available for *Sm* whose pathoadaptation to the CF lung is associated with populational genotypic and phenotypic heterogeneity in numerous phenotypical traits, i.e., growth rate, antibiotic resistance, biofilm formation, and both in vivo and in vitro pathogenicity [40,42].

More generally, an adapted, diversified population will encompass numerous sub-clonal variants that may vary on all characteristics listed within this review. The existence of these variants supports long-term survival of the pathogen in the CF lung, as it appears to quickly adapt to changing selective pressures through the selective advantage given to one or several of these variants by the new environmental conditions. This has previously been described as “the insurance hypothesis” [43].

## 4. Virulence Factors and Secretion Systems

**Figure 1:**
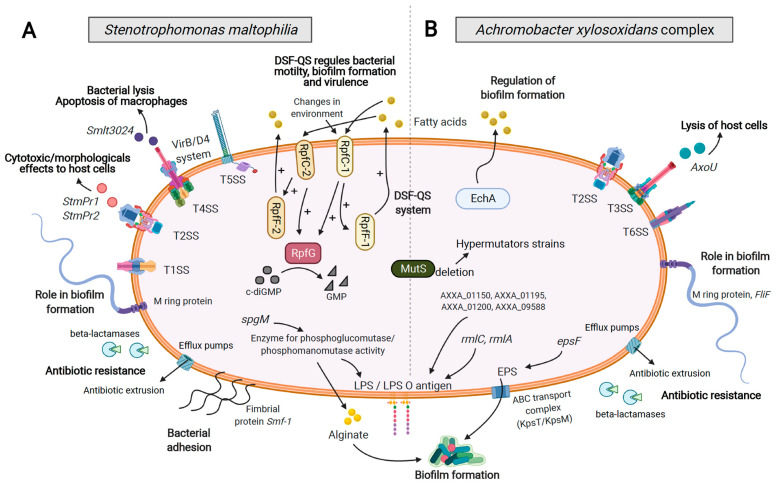
Schematic representation of major characteristics and mechanisms involved in colonization, persistence, and virulence of *Stenotrophomonas maltophilia* (**A**) and *Achromobacter xylosoxidans* (the most studied species in the *A. xylosoxidans* complex) (**B**): biofilm formation, antiobiotic resistance, hypermutation, secretion systems (TXSS: Type X Secretion System) and their associated molecules, Quorum Sensing (QS) system. In bold type: the role associated with each factor/mechanism. LPS, lipopolysaccharides; EPS, exopolysaccharides; DSF, diffusible signal factor (Figure created with BioRender.com, last accessed on 20 April 2021).

Bacteria possess specialized protein secretion systems allowing them to deliver a wide variety of proteins either in the extracellular space or directly into other cells. Classified into nine groups, from T1SS to T9SS (Type X Secretion System), these secretion systems are involved in the interaction between bacteria and their environment, a host organism, or other bacteria [50,51,52,53,54,55]. In the context of CF, these systems secrete effectors, allowing bacterial pathogens to escape from the host immune system but also to compete with other bacteria of the airway microbiota. Both *Axc* and *Sm* were shown to possess different secretion systems, common to many Gram-negative bacilli such as *Escherichia coli*, *B. cepacia* complex, or *P. aeruginosa* [51,56,57]. The TSSs harbored by *Axc* and *Sm* are shown in Figure 1.

First of all, *Axc* and *Sm* harbor T2SS, which contributes to virulence in many pathogens; as examples, T2SS-dependent substrates include the cholera toxin from *Vibrio cholerae* and exotoxin A from *P. aeruginosa* [51]. It may also be involved in host cell adhesion, biofilm formation, and intracellular infection of macrophages [58,59,60]. T2SS was identified in the sequenced and annotated genomes of *Axc* strain NH44784-1996 and strain Ax8 from CF patients [61]. *Sm* clinical strain K279a also possesses a T2SS that mediates the secretion of at least seven proteins and three proteolytic activities, and causes damage to lung epithelial cells, mostly through the secretion of serine proteases *StmPr1* and *StmPr2,* which induce cytotoxic and morphological effects on host cells [62,63].

Thirteen and twelve genes encoding for T3SS and T6SS respectively, were identified in the genome of *Axc* strain NH44784-1996 [61]. T3SS and T6SS mediate an effector transport by direct contact with the target cells [64] and T6SS has been shown to deliver lethal toxins into bacterial cells [65,66]. A complete T3SS locus has been observed in 72% of the 65 *Axc* genomes examined [67]. An important T3SS substrate is the phospholipase A2 AxoU, similar to the ExoU of *P. aeruginosa*, encoded by 98% of T3SS-positive *Axc* strains [67,68]. AxoU exhibits toxicity against eukaryotic cells by cleavage of membrane phospholipids, leading to lysis of the host cells, particularly phagocytic cells, and inhibits the innate immune response [67,68]. AxoU/T3SS-mediated cytotoxicity should thus be considered as an important virulence trait of *Axc*; however, further investigation is still required to fully elucidate this virulence mechanism, as bacterial internalization appears to be required for cytotoxicity [67].

A T4SS, homologous to the T4SS of the plant pathogens *Agrobacterium tumefaciens* and *Xanthomonas citri*, has also been identified in the genome of the clinical strain *Sm* K279a. T4SS mediates effector delivery in the extracellular medium or, in a contact-dependent manner, directly in the target cells, and has been involved in interbacterial antagonism and the delivery of lethal toxins into bacterial cells [64,65,66,69]. *Sm* K279a T4SS is encoded by a cluster of chromosomally located genes, *virB1-virB11* and *virD4*, called the virB/D4 system. Highly conserved among environmental and clinical strains of *Sm*, T4SS inhibits apoptosis in infected human epithelial cells, promotes apoptosis in infected mammalian macrophages, bacterial competition, and the death of multiple species, including *P. aeruginosa* [70]. In *Sm*, T4SS requires a direct contact with the target cells and the panel of affected cells, and its deleterious effects suggest that the VirB/D4 system is capable of interacting with a range of different receptors/membranes and then injecting diverse protein effectors into the target cells [70]. Among these, *Smlt3024*, an effector whose function is yet to be completely characterized, has shown the ability to reduce the growth rate of *E. coli* cells [69].

Finally, T1SS and T5SS have also been identified in the genome of the clinical strain *Sm* K279a [71].

Additionally, clinical *Axc* and *Sm* CF strains produce siderophores and secrete other virulence factors such as proteases, phospholipases, lipases, and nucleases, which contribute to host colonization and persistence through adhesion to and degradation of host tissues and iron uptake essential for bacterial growth [70,72,73,74,75,76].

## 5. Pathoadaptive Traits

### 5.1. Virulence Attenuation

Beyond chronic colonization, virulence attenuation is a pathoadaptive trait mostly described in *P. aeruginosa* (loss of T3SS and flagellum, reduced production of proteases, pyocyanins, and other virulence factors) [77] that was also reported for *Axc* and *Sm* CF strains. Regarding *Axc*, a strong decrease in the expression of genes coding for T3SS and motility [78], as well as mutations in virulence-associated genes [46], have also been observed for strains involved in late infection episodes compared with strains involved in early infections from the same patient. For *Sm*, a lower motility, a decrease or the loss of some virulence factors, and decreased virulence both in human cells and in an invertebrate model have been demonstrated [41,42]. All these observations underline that both *Axc* and *Sm* have the capacity to modulate their pathogenicity over time. Less exposed to the host immune system, this represents an additional key to bacterial persistence in the CF lung.

### 5.2. Adhesion, Motility, and Biofilm Formation

*Axc* isolates exhibited affinity to bind mucin, collagen, and fibronectin [79], while *Sm* was shown to be able to adhere to A549 respiratory epithelial cells and IB3-1 bronchial cells [80,81]. After adhesion, the ability to invade CF-derived IB3-1 bronchial epithelial cells was reported for *Sm* only, to date [81], and it represents a way to persist in the CF airways, as intracellular bacteria represent a less accessible reservoir of pathogens for both immune system and antimicrobial agents. Genes, such as the *smf*-1 gene coding for the fimbrial protein Smf-1, have a crucial role in the bacterial adhesion [82] and further biofilm formation as only *Sm* isolates containing the *smf*-1 gene were found to produce biofilm [83] (Figure 1). These observations are important for the initial CF patient airway colonization by *Axc* and *Sm* via adhesion to the surface of airway mucosae. Motility, particularly swimming motility, is also critical for this first step of adhesion and subsequent biofilm development and was found to be a constant characteristic of CF *Axc* and *Sm* strains [84,85]. Motility has indeed shown positive correlations with the *Sm* strains’ capacity for mucin-adhesion and biofilm formation [85] and the loss of flagella has been shown to significantly decrease *Sm* adhesiveness [81]. In *Axc*, more than 20 genes are involved in flagellum synthesis and most of them are downregulated during the sessile phase [76,86]. Among them, inactivation of the *flif* gene coding for the flagellar M ring protein leads to a reduction in biofilm formation [86]. This protein is also present in the flagellum of *Sm* and could have the same importance in biofilm formation (Figure 1). The early biofilm formation stages are thus mediated by flagella and fimbriae, allowing adhesion to epithelial cells [71].

After this initial pivotal stage of adhesion, both *Axc* and *Sm* have the ability to form biofilm, a highly organized multicellular structure in which bacterial cells are embedded in a self-produced extracellular matrix developing after transition of bacteria from their planktonic form to a sessile stage [14,79,84,87,88,89], with two distinct morphotypes observed in the CF lung for *Axc*, i.e., a surface-attached biofilm morphotype of small aggregates and an unattached biofilm morphotype of large suspended aggregates [90]. Congruent with previous genomic evidence [61], the ability to form biofilm has been observed in 97.1% to 100% of CF *Axc* strains [79,84,87] and is shared by several species of the *Axc*, i.e., *A. xylosoxidans* (*Ax*), *A. dolens*/*A. ruhlandii*, *A. insolitus*, *A. insuavis,* and *A. piechaudii* [79,84], with one third to 58% of isolates being strong biofilm producers. A slightly lower percentage of CF *Sm* strains are able to form biofilm, although this remains a characteristic presented by 87.5% to 90.2% of strains [91,92], with a high level of variability observed both among strains analyzed and in biofilm architecture [93]. This ability to form biofilm favors persistence and dissemination through the maturation of the biofilm and subsequent detachment of bacterial cells from the mature biofilm, in accordance with the general stages of biofilm development [94].

During the biofilm maturation phase, bacteria secrete various polysaccharides which form an exopolysaccharide (EPS) matrix in which they are embedded [95]. The main EPS secreted is alginate, which consists of mannuronic and glucuronic acid [96]. In *Axc*, the ABC transport complex (KpsT/KpsM) exports the polysaccharides through the cytoplasmic membrane [97]. According to Pompilio et al., the genes *spgM*, *rmlC*, and *rmLa* are associated with biofilm formation as well as 4 AXXA proteins (01150, 01195, 01200, 09588) [91] (Figure 1). In *Sm*, the *spgM* gene codes for a bifunctional enzyme with phosphoglucomutase and phosphomannomutase activity related to the metabolic process of carbohydrates and is involved in the lipopolysaccharide (LPS) and alginate biosynthesis, as observed for the homologous *algC* gene found in *P. aeruginosa* [89,98], whereas in *Axc*, *rmlC*, and *rmlA* genes, as well as Ax’s, AXXA-encoding genes are involved in the biosynthesis of the LPS O antigen [76,89] (Figure 1). It should be noted that mucoid phenotype related to EPS production is one of the most studied pathoadaptations of *P. aeruginosa* in CF but has been rarely reported in *Axc* and *Sm* subject to an accurate identification for older studies [99,100,101].

Numerous specificities are associated with the growth of biofilm (adaptation to hypoxic and acidic conditions, quiescent forms, high inoculum, gene expression modifications, etc.), all of which are important determinants for persistence. *Axc* and *Sm* undergo numerous metabolic and structural changes between the planktonic and sessile phases. The expression of the *epsF* gene coding for the *Axc* exopolysaccharides’ synthesis is increased by a factor of 6.4 during the sessile phase. In addition, the capsular ABC transporters of polysaccharides KpsT, KpsE, and KpsM are increased by 5.1 to 8.7 times [86]. For some *Axc* strains, a modification in the expression of genes involved in the stress response is also described during the sessile state. These genes code for diguanylate cyclase and phosphodiesterase synthesis [102]. In addition, an increase in the efflux pump activity during the sessile stage of *Axc* is observed compared to the planktonic stage. Indeed, the AxyA efflux pump regulated by the AxyAB-OprM operon has 7.4 times more activity in the sessile stage [86]. This efflux pump may be involved in the *Axc* biofilm metabolism, in addition to its role in antibiotic tolerance.

In *Sm*, factors identified as influencing biofilm formation are cell motility, genes involved in lipopolysaccharide/exopolysaccharide biosynthesis, SmeYZ and MacABCsm efflux pumps, iron availability, histidin kinase, the two-component signal transduction system BfmAK, and the diffusible signal factor (DSF) quorum sensing (QS) system [93,103,104]. This is supported and completed by recent transcriptomic analyses revealing that a small set of commonly regulated genes are involved in the biofilm lifestyle of *Sm,* with 6% to 9% of all genes being upregulated and 1% to 3% of all genes being downregulated in biofilms versus planktonic cells [93]. Commonly, upregulated genes show a large functional distribution, and they are mostly involved in transcription and translation followed by a remarkably high number of genes involved in iron acquisition, then in metabolism/biosynthesis, membrane proteins/transporters, and respiration/energy [93]. In addition, the authors identified an extracellular protease activity up to 40 times higher in *Sm* biofilm compared with planktonic cultures; however, although such an activity has previously been associated with virulence and biofilm formation, they could not link the increased protease activity observed with any particular virulence or biofilm-forming profile [93].

When looking at specific characteristics that might be presented by CF strains, the available studies showed no difference in biofilm formation between clinical and environmental *Sm* isolates [91], unlike *Axc* clinical strains which tend to produce more biofilm than environmental strains [90]. A focus on clinical strains has shown a similar proportion of *Axc* clinical isolates with the ability to form biofilm in CF and non-CF panels of strains [79]. For *Sm*, the proportion of CF and non-CF clinical strains able to form biofilm depends on the study, with some studies showing either no difference, congruent with the absence of difference in the presence of *rmlA*, *rpfF,* and *spgM* biofilm-associated genes [85,92], or a significantly higher proportion of biofilm-forming strains among non-CF strains [91]. In the latter study, the genes associated with biofilm formation, particularly the *spgM* gene, were differently expressed in *Sm* strains from CF and non-CF patients [91]. Nevertheless, CF *Sm* strains were shown to produce less biofilm biomass levels and exhibit a significantly higher proportion of biofilm-forming strains only when the moderate-biofilm production category was considered [91]. On the whole, they did not display any specific characteristics regarding their ability to form biofilm compared with other populations.

Considering the type of colonization, no difference in biofilm-forming capacity was observed between populations of *Axc* isolates from chronic or sporadic infection [84]. However, during persistence, a downward trend in biofilm biomass formed by clonally related, temporally isolated *Sm* and *Axc* strains from chronically infected CF patients was observed compared to the initial isolates obtained from the same patient as a consequence of *Sm* or *Axc* adaptation to the stressful lung environment [42,90]. For *Sm*, this was associated with a decrease in the biofilm’s ability to adhere and modifications in the biofilm structure that became distorted in the later infection stages compared with the early stages of infection where the *Sm* biofilm was well-structured with many juxtaposed layers [75].

Altogether, biofilm is an emblematic structure which allows *Axc* and *Sm* to survive under stressful conditions, making them able to persistently colonize the airways of CF patients. However, its production may greatly vary according to strains and conditions. Its regulation involves different structures such as flagella, fimbriae, efflux pumps, or transporters. Many metabolic changes and gene modulations take place during biofilm formation, with many genes activated or inhibited. Interestingly, similar pathways and genes were found involved in biofilm production in *Axc* and *Sm*. Finally, it should be pointed out that the biofilm structure confers a protection against various antimicrobial strategies, whether host defenses or antibiotic regimens [102], with *Axc* and *Sm* grown in biofilms exhibiting higher tolerance to various antibiotics [42,105,106]. Considered together with high levels of innate and acquired resistance presented by both pathogens (as presented below), antimicrobial tolerance conferred by the biofilm lifestyle adds to the difficulty of eradicating *Axc* and *Sm* through conventional antimicrobial strategies.

### 5.3. Antimicrobial Resistance

*Axc* and *Sm* have recently been shown to be more resistant than *P. aeruginosa* to airway antimicrobial peptides, with *Ax* being the most resistant species [107]. Both *Axc* and *Sm* are also innately multidrug-resistant (MDR) microorganisms able to increase their basal level of resistance through the acquisition of additional resistance mechanisms. In both species, resistance, either innate or acquired, is conferred by two major mechanisms: extrusion of the antibiotics through efflux pumps and enzymatic degradation (Figure 1).

A variety of efflux pumps and enzymes have been identified as contributing to antimicrobial resistance in *Axc* and *Sm* and are presented in Table 1.

Considering antibiotics, the most widely used in the management of bacterial infections, resistance in *Axc* affects most cephalosporins except ceftazidime via the production of an inducible chromosomal β-lactamase, aztreonam, and aminoglycosides, while *Sm* is usually resistant to all β-lactams except ticarcillin-clavulanate and ceftazidime via the production of two inducible β-lactamases, including a carbapenemase (metallo-β-lactamase L1), aminoglycosides, and colistin. Antibiotics available to treat MDR *Axc* and *Sm* infections are thus limited, with co-trimoxazole usually considered as the best option for treatment when patients meet the criteria for treatment initiation [147,148,149]. Otherwise, therapy should be guided by antimicrobial susceptibility testing results. For *Axc*, the most active agents beside co-trimoxazole are minocycline, meropenem, or imipenem, piperacillin-tazobactam, ceftazidime, and/or chloramphenicol, depending on the study [150,151,152,153]. For *Sm*, chloramphenicol, minocycline, or doxycycline can be considered for the treatment, as well as ticarcillin-clavulanate, ceftazidime, and levofloxacin, for which clinical breakpoints are available. However, the use of both first-line and second-line treatments may be compromised when infection involves exogenous strains which have acquired additional resistance up to being extensively drug-resistant or strains with in vivo acquisition of resistance during chronic colonization [31,37,42,78,79,112,113].

Due to the highly selective surrounding conditions encountered by pathogens in the CF lung, including significant exposure to antibiotics, it should be noted that bacterial populations displayed specific resistance characteristics. One recent study showed that *Sm* CF strains were more resistant to piperacillin-tazobactam and to cotrimoxazole compared to non-CF strains, with MDR strains being significantly more prevalent in CF and hypermutable CF strains showing a high number of antibiotic resistances [92]. In addition, it is worth noting that small-colony-variants (SCV) of *Sm* have been identified in samples of CF patients [49,154] and that SCV phenotype can be selected during the selective pressure of the first-line cotrimoxazole antimicrobial therapy [154]. SCV morphotype is usually associated with slow-growing subpopulations of bacteria displaying distinctive phenotypic and pathogenic traits of which increased resistance to antibiotics and contribute to persistent or recurrent infections.

Altogether, antimicrobial resistance makes the antibiotic treatment of *Axc* and *Sm* infections challenging in CF patients, particularly if we consider that both pathogens may also be protected from antimicrobial drugs in the biofilm formed in the CF lung, where higher antibiotic minimal inhibitory concentrations are usually observed compared with the planktonic forms [105,106].

## 6. Competitive Interactions

Whereas *P. aeruginosa* and *S. aureus* have both been greatly studied for their interspecies competitive behavior in the context of CF, *Axc* and *Sm* have received far less attention despite their increased prevalence in a restricted niche represented by CF lungs and their frequent co-isolation with other pathogens, suggesting that they also have to compete for space and nutrients, just like other members of the resident microbial community [155]. Several of the *Axc* and *Sm* characteristics listed above, especially biofilm formation, are also well-known traits influencing competitive or cooperative bacterial interactions [156].

A few studies included *Sm* CF clinical strains and have revealed complex, reciprocal interferences with *P. aeruginosa* CF clinical strains or reference strains. Most studies revealed that *P. aeruginosa* inhibited *Sm* growth but that it confers to *Sm* an increased tolerance to antibiotics, such as ciprofloxacin and tobramycin within dual biofilms. In return, *Sm* displays the ability to modulate *P. aeruginosa* virulence in mixed biofilms [23,155,157,158]. These observations support the existence of a cooperative pathogenicity between the two species, as proposed by Pompilio et al. [158]. These interactions appear to require a direct contact between *Sm* and *P. aeruginosa* as *Sm* growth inhibition was not observed when *P. aeruginosa* culture supernatant was tested, suggesting that *P. aeruginosa* T6SS was involved [159]. Other modifications highlighted during *Sm*–*P. aeruginosa* co-cultures are modifications in *P. aeruginosa* motility and pigment production [157,158]. Altogether, about one third of the *Sm*–*P. aeruginosa* pairs of strains tested (31%, 18 of 59) displayed at least one modification affecting growth, motility, and/or pigment production when tested in co-culture compared with the corresponding monocultures [157]. Divergent competitive ability was, however, observed according to the study conditions regarding dual biofilm biomass and *P. aeruginosa* motility, either decreased or increased [155,157,158], at least partly linked to the tested strains, i.e., reference or clinical strain, CF or non-CF isolates, time to first colonization for CF isolates, and the planktonic or sessile growth conditions of the assays. Pompilio et al. identified several *P. aeruginosa* virulence genes whose expression was significantly modified by co-culture with *Sm*, with protease and alginate-encoding *aprA* and *aglD* genes being upregulated and the QS-related *rhlR* and *lasI* genes being downregulated [158]. A first recent in vivo study has brought new knowledge of cooperativity between *Sm* and *P. aeruginosa,* showing the co-localization of both pathogens in the lungs of BALB/cJ mice and the integration of *Sm* in *P. aeruginosa* biofilm, both observations being factors favoring interaction between the two species. Increased *Sm* loads, correlated with *P. aeruginosa* loads, were observed in lung homogenate and broncho-alveolar lavage fluid samples in the presence of *P. aeruginosa* [23]. Viable *P. aeruginosa* cells were necessary to confer a significant persistence benefit to *Sm,* showing that this persistence was not only mediated by passive protection in biofilm but also required an active cellular process [23].

Regarding the interaction between *Sm* and other species, *Sm* motility and/or growth alterations have occasionally been observed in *Sm*–*Axc* and *Sm*–*S. aureus* co-cultures [157]. *Sm* can also establish mixed biofilm with *Aspergillus fumigatus* which can be co-isolated from the airways of CF patients [160]. In *Sm*–*A. fumigatus* co-culture biofilms, the morphology of *A. fumigatus* changes and its growth decreases, whereas antimicrobial susceptibility of both partners is modulated (increased *A. fumigatus* susceptibility to amphotericin B and increased *Sm* tolerance to levofloxacin) [161,162]. In poly-species culture, *Sm* has been shown to confer some protection to *Burkholderia cenocepacia* against hydrogen cyanide, a toxic metabolite released by *P. aeruginosa* [163]. Finally, *Sm* also demonstrates the ability to lyse CF bacterial competitors such as *P. aeruginosa* using T4SS [70] or to disrupt the hyphal transition and biofilm formation of *Candida albicans* through its DSF QS system [164].

The competitive ability of *Axc* has been even more rarely studied than that of *Sm*. The first evidence of *Axc*’s competitive ability revealed that nearly half of the *Axc*–*P. aeruginosa* pairs of strains tested (49%, 26/53) displayed at least one modification affecting growth, motility, and/or pigment production when tested in co-culture compared with the corresponding monocultures [157]. Some *Axc* clinical strains were able to outcompete clinical as well as environmental strains of *P. aeruginosa* by inhibiting the above major *P. aeruginosa* pathophysiological traits. In addition, *Axc* has the ability to outcompete *Sm* by decreasing its growth and/or motility. Finally, interactions affecting *Axc* motility were also observed on an intra-species level, with one *Axc* strain being able to influence the motility of another, even when co-isolated from a unique sample [157].

As previously recognized for major CF pathogens, interactions involving emerging CF pathogens appear complex and varied and probably act synergistically. Bacterial competition affects the major bacterial pathophysiological traits represented by growth, motility, biofilm formation, antimicrobial tolerance, and/or *P. aeruginosa* pigment production, considered as an indirect marker of *P. aeruginosa* virulence. However, additional studies using well-documented clinical strains and in vivo models are still needed to better delineate the importance of these interactions in shaping the CF lung polymicrobial community and decipher the mechanisms involved in these interactions.

## 7. Quorum Sensing Regulation

Bacterial populations use quorum sensing (QS) molecules for intercellular signaling [165], which allow coordination of gene expression when they reach a critical cell density. QS is a unique regulatory mechanism that acts through the production, detection, and response to extracellular signaling molecules called autoinducers. Autoinducers accumulate in the environment as bacterial populations become concentrated. When they reach a high local concentration, the cells “detect” the change in their number and the population responds with coordinated expression of specific target genes [165,166]. This coordination allows the bacterium to regulate various mechanisms involved in colonization or virulence, such as biofilm formation, motility, and the production of siderophores and other virulence factors. This system strongly contributes to the bacterial establishment and persistence in the host and is long recognized as a key parameter in bacterial virulence and pathogenesis. Indeed, pathogens with QS pathways are usually considered as able to infect host organisms more effectively. With regard to bacteria that utilize QS as part of their pathogenic lifestyle, *P. aeruginosa* is the best understood in terms of the virulence factors regulated and the role quorum sensing plays in pathogenicity. Despite increased knowledge presented hereafter for *Sm* and *Axc*, research is still needed to decipher the role that QS plays in their pathogenicity in CF patients.

### 7.1. Diffusible Signal Factor (DSF) System

This QS system is present in various Gram-negative bacteria, including *P. aeruginosa* [165]. It is also characterized in strain K279a, a reference *Sm* strain. The QS system is based on the diffusible signal factor (DSF), with cis-11-methyl-2-dodecenoic acid as the main signaling factor [167]. Its production is controlled by the *rpf* (regulation of pathogenicity factors) cluster, and two types of *rpf* clusters have been demonstrated in clinical strains: *rpfF*-1 (60%, 47 of 78 strains) and *rpf*-2 (40%, 31 of 78 strains), with established links between the type of DSF-based QS system and the phenotypes of biofilm formation, virulence, and antimicrobial resistance of the strains [168,169]. *rpf*-1-type *Sm* strains basically produce DSF in response to diverse environmental changes, whereas *rpf*-2-type *Sm* strains are deficient for basal DSF production while retaining their ability to detect DSF produced by other strains and subsequently produce DSF [168]. The *rpf* cluster encodes for RpfF synthase (which has different N-terminal regions according to the *rpf* cluster type) and the RpfC/RpfG two-component system responsible for perception and transduction of the DSF. Activated RpfG converts cyclic diguanylate monophosphate (c-di-GMP) to GMP, thereby controlling the expression of genes, which regulate biofilm formation, virulence, and bacterial motility in *rpfF*-1 strains [170] (Figure 1). DSF-mediated regulation has been associated with a decrease in motility, siderophores, protease level, and virulence in different models [170,171], and either with an increase [168] or a decrease [171] in biofilm production, depending on the study. In the *Sm rpf*-2 system, RpfC-2 blocks RpfF-2, which in turn stops DSF synthesis. Exogenous DSF signals released by surrounding bacteria (e.g., *rpf*-1 strain) are detected by RpfC-2, liberating active RpfF-2 to produce DSF (Figure 1), thus stimulating bacterial virulence, as demonstrated by experiments in the zebrafish model [172].

Other than its regulatory role, the *Sm* DSF system plays a role in inter-microbial interference and competition and, as stated above, has been shown to disrupt the hyphal transition and biofilm formation of *C. albicans* [164].

With regard to *Axc* QS systems, nothing had ever been described before the study by Cameron et al., showing that Enoyl CoA hydratase (echA) plays a central role in the biosynthesis of cis-2 fatty acid signaling molecules by *A. xylosoxidans* (Figure 1). As described in *Sm*, these molecules are DSF, which regulate biofilm formation and tolerance to antimicrobials [102].

### 7.2. Other Quorum Sensing Factors

A small protein Ax21 was first described as a QS-related virulence factor in *Xanthomonas* species pathogenic for plants. Then, later identified in clinical strains of *Sm*, its production was linked with several important pathophysiological traits in *Sm*, such as virulence (both in zebrafish and larval *Galleria mellonella* infection models), antibiotic tolerance/resistance, biofilm formation, and motility [173,174,175]. Secreted within the outer membrane vesicles, Ax21 is thought to mediate intraspecies communication. However, as synthesis and secretion of Ax21 can be influenced by the DSF-QS system, further investigations are required to find out whether Ax21 acts indirectly through the influence of the DSF [176]. Finally, although *Sm* does not have a complete Acyl Homoserin Lactone (AHL)-mediated QS system (LuxI/LuxR family) like *P. aeruginosa*, it possesses the LuxR regulator element of this system, known as SmoR [177]. This LuxR solo SmoR confers *Sm* the ability to detect AHL signals produced by other microorganisms, with AHL produced by *P. aeruginosa* being able to increase the swarming motility of *Sm,* for example, and thus represents another way for interspecific communication [177].

## 8. Conclusions

A review of the research on *S. maltophilia* and *A. xylosoxidans* complex shows that both emerging pathogens in CF share important pathophysiological features with other well-known CF pathogens such as *P. aeruginosa*, making them important members of the complex bacterial community living in the CF lung and successful CF pathogens. Indeed, the summarized literature highlights that both *Sm* and *Axc* are well-armed for the colonization and subsequent persistence in the hostile environment represented by the CF lung, as they harbor a large panel of adaptive strategies allowing them to face adverse, fluctuating conditions. Most of the pathogenic phenotypes described in this review are interconnected with each other, either positively or negatively, and are subjected to rapidly changing surrounding selective pressures in the host. Their evolutionary dynamics are thus highly complex, intimately linked to the patient airway colonization history and the specific host/pathogen partnership shaping the progress of bacterial pathoadaptation in a bacteria–host evolutionary arms race. In this context, the increased knowledge of major pathophysiological traits presented by both *Sm* and *Axc* and their regulation by QS systems opens up new perspectives for the care of CF patients, with novel antimicrobial strategies targeting either directly *Sm* or *Axc* characteristics (anti-biofilm) or interfering with their QS communication signals (quorum quenching) as an anti-virulence strategy [75,149,178,179,180].

## Figures and Tables

**Table 1:** Main antibiotic resistance determinants in *Achromobacter xylosoxidans* complex and *Stenotrophomonas maltophilia* and their contribution to intrinsic or acquired antimicrobial resistance.

Bacteria/Types of Mechanisms	Resistance Mechanisms	Spectrum	Type of Resistance	Reference
***A. xylosoxidans* complex **				
Efflux pumps	AxyABM	Cephalosporins (except cefuroxime and cefepime), aztreonam	Int	[108]
	AxyXY-OprZ	Aminoglycosides, tetracyclines including tigecycline, fluoroquinolones, erythromycin, cefepime, carbapenems	Int	[109,110]
	AxyEF-OprN	Some fluoroquinolones, tetracyclines, carpabenems	Int?	[86]
β-lactamases	OXA-114	Piperacillin, ticarcillin, benzylpenicillin, cephalothin	Int	[111]
	ESBL (CTX-M, VEB-1); AmpC (CMY-2, AmpC)	All β-lactams except carbapenems	Acq	[112,113,114]
	Plasmidic (IMP, VIM and KPC) and chromosomal carbapenemase (TMB-1)	All β-lactams except aztreonam (VIM-2 strains resistant to aztreonam)	Int and Acq	[115,116,117,118]
Others	*aac*(6′)Ib-cr, *qnrA*, *oqxA*, *oqxB*	Fluoroquinolones, aminoglycosides	Acq	[113,119]
	*gyrA*, *parC*	Fluoroquinolones	Acq	[120]
***S. maltophilia***				
Efflux pumps	SmeABC	Aminoglycosides, β-lactams, fluoroquinolones	Acq	[121]
	SmeDEF	Tetracycline, chloramphenicol, macrolides, fluoroquinolones, sulfamethoxazole, trimethoprim, trimethoprim/sulfamethoxazole, tigecycline	Int and Acq	[121,122,123,124]
	SmeGH	Fluoroquinolones, β-lactams, tetracycline, polymyxin B	Int and Acq	[71,125]
	SmeIJK	Aminoglycosides, tetracycline, minocycline, ciprofloxacin, levofloxacin	Int and Acq	[126]
	SmeOP	Nalidixic acid, doxycycline, aminoglycosides, macrolides	Int	[127]
	SmeVWZ	Quinolones, chloramphenicol, trimethoprim/sulfamethoxazole	Acq	[124,128,129]
	SmeYZ	Aminoglycosides, tetracycline, trimethoprim/sulfamethoxazole	Int and Acq	[103,129,130]
	MacABCsm	Aminoglycosides, macrolides, polymyxins	Int	[131]
	EmrCABsm	Nalidixic acid, erythromycin	Int	[132]
	FuaABC	Fusaric acid	Int	[133]
	SmrA	Fluoroquinolones, tetracycline	Int and Acq?	[123,134]
β-lactamases	L1 Class B3 Zn2+ -dependent metallo-β-lactamase	β-lactams (except monobactams)	Int	[135,136]
	L2 Class A clavulanic acid- susceptible cephalosporinase	β-lactams	Int	[135,137]
	TEM-2 penicillinase	Ampicillin, piperacillin	Int	[138]
	CTX-M-1 β-lactamase (ESBL)	β-lactams	Acq	[139]
Others	*aac*(6′)-Iz, *aph*(3′)-IIc, *aac*(6′)-Iak	Aminoglycosides	Int	[140,141,142,143]
	Smqnr	Quinolones	Int and Acq	[124,144,145,146]

Int, intrinsic resistance; acq, acquired resistance; ?, suspected role in intrinsic or acquired resistance. Bacterial names are indicated in bold on a grey background.

## Data Availability

Not applicable.
